# The Epidemiology of Salivary Glands Pathologies in Adult Population over 10 Years in Poland—Cohort Study

**DOI:** 10.3390/ijerph19010179

**Published:** 2021-12-24

**Authors:** Michał Żurek, Anna Rzepakowska, Kamil Jasak, Kazimierz Niemczyk

**Affiliations:** 1 Department of Otorhinolaryngology Head and Neck Surgery, Medical University of Warsaw, 1a Banacha Str., 02-097 Warsaw, Poland; kniemczyk@wum.edu.pl; 2 Doctoral School, Medical University of Warsaw, 61 Żwirki i Wigury Str., 02-091 Warsaw, Poland; 3 Students Scientific Research Group at the Department of Otorhinolaryngology Head and Neck Surgery, Medical University of Warsaw, 1a Banacha Str., 02-097 Warsaw, Poland; s071767@student.wum.edu.pl

**Keywords:** salivary gland pathologies, epidemiology, Poland, incidence rate

## Abstract

Background: The aim of this study was a comprehensive analysis of the incidence of different salivary gland pathologies in the adult population of Poland. Methods: A retrospective analysis of salivary gland pathologies diagnosed in Poland in 2010–2019 based on the National Health Fund (NHF) database was performed. Non-neoplastic diseases, and benign and malignant lesions were identified using ICD-10 codes. Demographic characteristics, incidence rates, and the number of inpatient and outpatient medical services were analyzed. Results: Salivary gland pathologies were diagnosed in 230,589 patients over 10 years (85.5% were non-neoplastic lesions, 11.53% benign and 2.93% malignant neoplasms). Incidence rate for all pathologies was 59.94/100,000. The mean incidence for malignant neoplasms was 1.78, and decreasing trend was observed over the analyzed period. Contrarily, for benign neoplasms (mean incidence—6.91), an increase in numbers was noted annually. The incidence for non-malignant lesions was quite stable (mean: 51.25) over the time. The highest number of medical services per patient concerned malignant neoplasms (on average, two hospital stays, and eleven outpatient consultations). Conclusions: An increase of benign salivary gland tumors, and a decrease of malignant neoplasms was observed during the studied period. The number of medical services related to salivary gland pathologies increased during the period under study.

## 1. Introduction

Salivary gland pathologies are a heterogeneous group of diseases with diverse clinical manifestations, and a heterogeneous pathomorphological image. In clinical practice, effective diagnosis and early treatment of patients with neoplastic lesions are the most important ways to achieve a good therapeutic effect. Inflammatory lesions of the salivary glands, especially of the acute or subacute course, are associated with more severe pain in patients, but the prognosis is favorable. Unfortunately, a number of inflammatory and reactive lesions of the salivary glands may mimic neoplastic disease, so in most patients, imaging diagnosis, including ultrasonography and magnetic resonance imaging or computed tomography, must be routinely performed. Salivary gland tumors account for 3 to 10% of all head and neck cancers [1,2,3,4]; in Europe, this percentage is 8.5% [5]. Most of the proliferative lesions in the salivary glands are benign, and only less than 20% are malignant [6]. According to published data, the incidence is 0.4–13.5 per 100,000 inhabitants [1,2,5,7]. The incidence of salivary gland tumors depends on age and geographical area. The average incidence increases from 0.15 for patients under 25 years of age, to 1.2 for those in age range of 25–64 years, up to as high as 4.3 per 100,000 in the population over 65 years [5]. The highest incidence of salivary gland cancer occurs in European and North American populations compared to other continents’ inhabitants [4].

Most of the available epidemiological studies on the pathology of the salivary glands focus selectively on the group of neoplastic diseases, with the largest number of studies analyzing the occurrence of malignant lesions [1,2,3,4,5]. Among the publications, only few are cohort studies; observations from one or several research centers predominate. Pathologies of salivary glands of inflammatory etiology are less frequently described in the literature, and the epidemiology of this type of lesions is scarcely analyzed in comparison to neoplasms. It is estimated that the prevalence of inflammatory lesions of the salivary glands in the population is about 1.2%, but in most cases, the disease is asymptomatic or scantly symptomatic [8]. Inflammatory changes associated with salivary gland stones are responsible for up to half of such pathologies [9].

In the present study, we performed a comprehensive analysis of the incidence of different types of salivary gland pathologies, including non-neoplastic lesions, and benign and malignant neoplasms in the adult patient population in Poland over the period from 2010 to 2019. The aim of the study was to comprehensively evaluate the epidemiology of different types of salivary gland pathologies, and the trends in incidence and prevalence.

## 2. Materials and Methods

The research project is a retrospective analysis of data from the National Health Fund (NHF) [10]. In Poland, health care is based on insurance, and supplied by the NHF, a publicly funded health-care system. The system is free for all insured citizens, employees, registered unemployed persons, and spouses or children of an insured person. The NHF database includes primary, outpatient, and inpatient care, so the data in the analyses refer to all patients in Poland receiving healthcare financed from public funds. The information in NHF databases include medical data and demographic characteristics of the patients, in particular, gender, age, and place of residence. Diagnoses are coded according to the International Statistical Classification of Diseases and Related Health Problems ICD-10 (ICD-10). The research group was defined as patients diagnosed with salivary gland pathologies between 2010 and 2019. Salivary gland pathologies are defined by the following ICD-10 codes, and divided into the following three groups (Table 1):

- Malignant neoplasms: C07, C08, C08.0, C08.1, C08.8, C08.9
- Benign neoplasms: D11, D11.0, D11.7, D11.9
- Non-neoplastic diseases: K11.0, K11.1, K11.2, K11.3, K11.4, K11.5, K11.6, K11.7, K11.8, K11.9

For each salivary gland pathology group, the mean age of patients at the diagnosis with standard deviation, and the percentage of males and females were calculated. The incidence of each salivary gland pathology group was calculated by dividing the number of new patients by 100,000 adult citizens. Data on the number of adults in Poland are from Statistics Poland [11], and include all citizens over 18 years of age, regardless of their insurance status with the NHF. The number of outpatient consultations and hospital stays for patients with salivary gland diseases was also analyzed. The last section of the study presents a map of the provinces with the number of treated patients per 100,000 inhabitants for different types of lesions.

## 3. Results

In the analyzed period, pathologies of salivary glands were diagnosed in 230,589 patients. The percentages amounted to 85.5% for non-neoplastic lesions, 11.53% for benign tumors, and 2.93% for malignant neoplasms.

### 3.1. Incidence Rates and Characteristics of Patients

**Figure 1:**
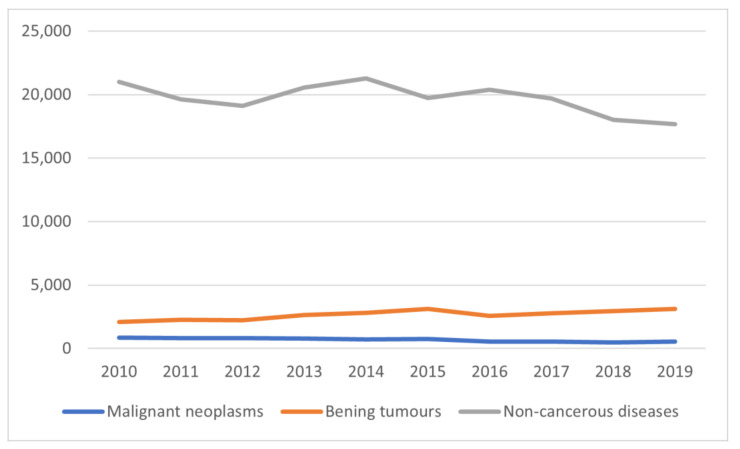
Overview of new salivary gland pathologies diagnosed in 2010–2019 in Poland.

The incidence rate for all types of salivary gland pathologies was 59.94/100,000, including malignant neoplasms—1.78, benign neoplasms—6.91, and non-malignant lesions—51.25 (Table 2). Over the 10 years, there was a decrease in the incidence of malignant neoplasms (from 2.198 in 2009, to 1.449 in 2019; the absolute number of new patients decreased on average by 4.57% per year). The downward trend was also observed for incidence of non-malignant pathologies (from 54.514 in 2009, to 46.091 in 2019; 1.89% decrease in new patients each year), whereas the number of non-malignant neoplasms gradually increased (from 5.398 in 2009, to 8.137 in 2019; average annual increase in new patients of 4.62%). The incidence rates and demographic characteristics of the studied group are presented in Figure 1 and Table 2.

Overall, the mean age was highest in the group of patients with malignant salivary gland tumors—62.2 years. Moreover, the progressive increase of the mean age for this patient category was observed, on average by 0.34 per year. A slight predominance of incidence in males (56.70%) was found in this group. Patients with benign salivary gland tumors were younger, with an average age of 55.27 years at the time of the diagnosis. Also, in this group, an increase in the mean age value was observed, with an average of 0.45 per year. The incidence was predominant in women—55.02%, but over the study period, the percentage advantage decreased by 2.88%. The lowest mean age was found for non-neoplastic salivary gland pathologies—50.23 years, but the standard deviation was the highest in this group of patients (±21.05 years). The vast majority of patients in this group were women (61.2%), and this proportion did not change over the study period.

The comprehensive analysis of newly diagnosed salivary glands pathologies with respect to all analyzed ICD 10 codes is summarized in Supplementary Materials Table S1, allowing the comparison of diseases incidence within major salivary glands, and some differentiation of the diagnosis for non-neoplastic lesions.

In terms of location, malignant neoplasms predominated in the parotid gland—C07 (4469 of 6844 total patients, 65.30%), and the number of new cases for this location decreased on average by 4.13% each year. Among non-malignant neoplasms, parotid lesions (D11.0) also had the highest share (12,259 of 26,601 patients, 46.08%). An average annual increase in the number of new lesions in this location of 10.32% was observed. A similar trend was also observed for benign tumors of the sublingual and submandibular salivary glands (D11.7 and D11.9), and a mean increase in the number of patients by 7.61% was calculated. For non-neoplastic pathologies, the most common diagnosis was general salivary gland diseases—K11 (128,845 patients, 65.36%), followed by salivary gland inflammation—K11.2 (24,302 patients, 12.33%), and sialolithiasis—K11.5 (12,265 patients, 6.22%). There was also an increase in the number of diagnoses for salivary gland inflammation and sialolithiasis, which annually reached on average 7.21% and 6.41%, respectively.

### 3.2. Outpatient and Inpatient Care

**Figure 2:**
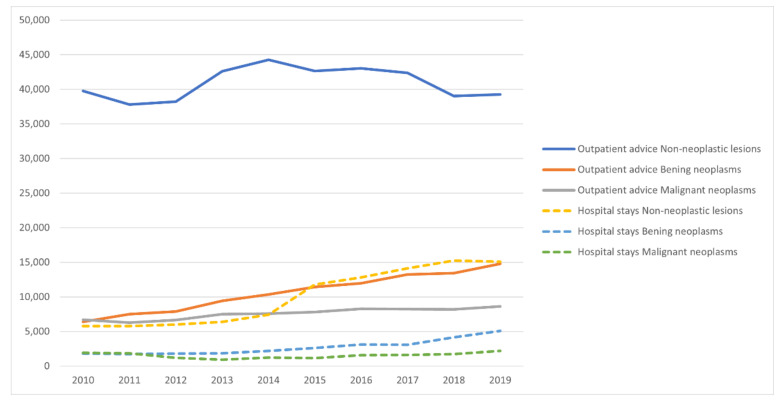
Number of medical services related to salivary gland pathologies in Poland between 2010 and 2019.

Data on outpatient and inpatient services related to salivary gland pathologies are presented in Table 3 and Figure 2. The number of outpatient services related to non-malignant salivary gland pathologies changed little between 2010 and 2019 (from 38,217 to 44,267), whereas a higher than 2.6-fold increase was noted for hospital stays in this group of patients (from 5786 to 15,073). On average, each patient with such pathology had two visits at the outpatient clinic, and every second patient was admitted to hospital for this reason. In the case of benign salivary gland tumors, the number of outpatient consultations increased 2.3-fold (from 6411 to 14,803), and the average patient with a benign tumor had four outpatient consultations. The number of inpatient stays for benign tumors also increased from 1800 to 5076 (a 2.8-fold increase). Patients with malignant neoplasms required the highest number of outpatient consultations and hospital stays, respectively: 11 outpatient services per patient, and more than 2 hospital stays. There was a small increase in outpatient visits in this group (about a 1.3-fold rise), whereas the number of hospital stays increased from 911 in 2013, to 2197 in 2019.

### 3.3. Regional Prevalence

**Figure 3:**
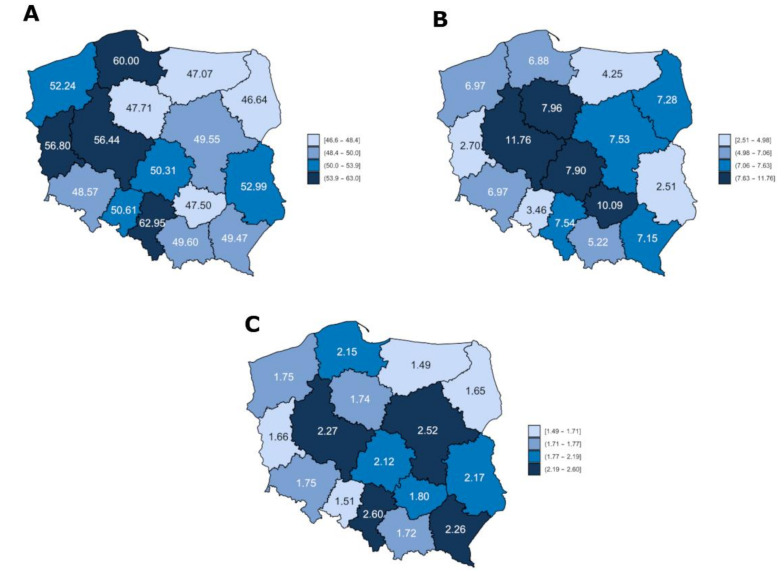
Prevalence of salivary gland pathologies by voivodeships in Poland in 2010–2019 per 100,000 inhabitants. (**A**)—Prevalence of non-neoplastic salivary gland diseases; (**B**)—Prevalence of benign salivary gland neoplasms; (**C**)—Prevalence of malignant salivary gland neoplasms.

Figure 3 presents the mean prevalence (per 100,000 inhabitants) of salivary gland pathologies in the analyzed period divided into voivodeships in Poland. Territorial variation of prevalence was observed for all types of lesions. In cases of non-neoplastic pathologies and benign neoplasms, the highest prevalence was observed in the Greater Poland Voivodeship. The highest prevalence of malignant salivary gland tumors was observed in the Lower Silesian region. It is worth noticing that the prevalence of salivary gland benign lesions varies greatly, from 2.51 for the Lublin Voivodeship, to 11.76 for the Greater Poland Voivodeship (Figure 3B).

## 4. Discussion

The presented epidemiological analysis of salivary gland pathologies among adult Poles presents extensive and comprehensive data from the last decade, which may be used in the prognosis and planning of medical services, and constitute a basis for further research on the observed variability of incidence depending on the region of the country.

In terms of gender predisposition, our analysis is consistent with the results of earlier, smaller population-based studies conducted by other Polish authors: women constituted about 55.7–56.5% of patients with benign salivary gland tumors, and 47.6–49% of patients with malignant tumors [12,13]. Data from other countries are more varied, and the percentage of women among patients with malignant lesions of the salivary glands ranges from 46% to 52% [2,14,15,16,17,18].

On the basis of performed analyses concerning the mean age of incidence of particular salivary gland pathologies, differences were observed depending on the type of lesions, and over the years in the analyzed period. Other Polish studies report the mean age of patients with benign salivary gland lesions in the range 50.1–52.63 years, and with malignant lesions in the range of 59.4–65.1 years [12,13]. However, this study included patients from earlier years, and as suggested by the results of the current analysis, there was an annual increase in the mean age of onset for benign and malignant pathologies observed. The variation in the mean age of onset also shows marked differences between countries. The studies report the mean age of patients with malignant lesions of the salivary glands between 51–62 years [2,14,15,16,19].

The incidence of salivary gland pathologies shows differences in the examined period; however, the average values are similar to those reported in other countries. Bradley et al. [7], in a study from Great Britain, reported similar incidence values of 6.2–7.2 for benign, and 0.83–1.38 for malignant neoplasms per 100,000 inhabitants. The incidence of salivary gland malignancies varies from 0.5 to 2 per 100,000 inhabitants, with the highest value for Croatia, and the lowest for Japan [16,19,20,21]. It is worth noting that the changes in incidence rates depend on the nature of the lesion, with an increasing trend for benign salivary gland tumors (from 5.4 to 8.2), and a decreasing trend for malignant tumors (from 2.2 to 1.4) between 2010 and 2019. A similar incidence trend for benign salivary gland tumors was observed in the work of Stryjewska-Makuch et al. [3]. In turn, these results differ from those presented in the Danish study by Westergaard-Nielsen et al. [19], where an annual increase in the incidence of salivary gland malignancies was observed, but the analysis included earlier years, from 1990 to 2015. A similar increase in the incidence of malignancies was reported in the United States at the turn of the century between 1973 and 2009 [22].

The observed reverse trends in the incidence of benign and malignant salivary gland tumors in the analyzed study can be explained with more widespread and available imaging diagnosis, especially the ultrasounds in recent decades. Therefore, the benign salivary gland pathologies are identified and treated earlier, and the group of benign lesions with the potential for malignant transformation has been currently reduced.

In the analyzed material, neoplastic pathologies of salivary glands were most frequently located in the parotid glands. Malignant tumors of the parotid gland constituted 65.3% of all diagnoses in this group. Analyzing all parotid neoplasms, benign lesions constituted 73.28%, and malignant ones constituted 26.72%. In previously presented studies from different centers in Poland, similar percentages of malignant lesions were observed [12,13], although in some studies, due to lower heterogeneity or a more specialized center profile, the percentages of parotid gland involvement for malignant neoplasms were overestimated, even up to 79.6% [23]. In a study from Israel, salivary gland malignancies initially occupied the parotid glands in 55% of cases. Similarly, in a population-based study from Denmark, the localization of malignant neoplasm in the parotid gland was reported in 51.8–52.5% of cases, and in a study from Sweden, this percentage was 57.5% [24]. In contrast, a study by Tian et al. [18] from China reported only 34.12% of malignant tumors in the parotid gland.

The strength of the following study is the analysis of medical services related to salivary gland pathologies. The highest number of services per patient concerns those with malignant neoplasms (an average of 11 outpatient specialist care visits per patient, and more than 2 hospital visits). At the same time, there has been an annual increase in outpatient visits for this group of patients between 2010 and 2019, indicating improved care for oncological patients. For benign salivary gland tumors, there has also been an increase in outpatient services (2.3-fold), as well as inpatient services (2.8-fold). Inpatient treatment is also increasing among patients with non-malignant salivary gland lesions (2.6-fold increase), which may be related to more favorable billing for surgical procedures such as sialoendoscopy during the hospitalization. The upward trend in the number of outpatient services may result from the release of limits on services, and from an increase in health awareness in the population. The increase in the number of inpatient services, especially in the case of non-cancer diseases, is also associated with the development of diagnostic and treatment methods, such as sialoendoscopy, which are preferably carried out in hospital conditions. Comparison of the frequency and structure of services with other countries would be an interesting aspect and a pretext for the assessment of the effectiveness; unfortunately, there was no publication presenting such data found.

Another very interesting observation from the study is large regional variation in the prevalence of salivary gland pathology in individual provinces in Poland. On the one hand, the highest rates were noted in provinces with the leading clinical head and neck surgery centers that have extensive experience with complex salivary gland pathologies. Unfortunately, these areas are also highly urbanized and industrialized on a national scale, which may be a factor of exposure for the inhabitants. Similar observations regarding the large regional variation in the prevalence of salivary gland malignancies were presented in the study by Kordzińska-Cisek et al. [4].

The presented analysis has some limitations related to the lack of clinical data, complete histopathological diagnoses, and stage of malignant lesions, which are not reported to the NHF, and thus, could not be included in the study. The reason for these limitations is primarily the fact that the data in NHF database are mainly recorded for administrative purposes, not for research.

## 5. Conclusions

The analysis revealed an increasing trend in the incidence of benign salivary gland neoplasm, and a decrease in malignant pathologies over the recent decade in the Polish population. The number of services for patients with benign salivary gland neoplasms has increased in the period under review, and further increases should be expected. Although there was an observed decrease in the number of malignant neoplasms, there was also an increase in the number of outpatient services noted in this group. At the same time, the number of non-malignant salivary gland diseases is decreasing from year to year, but the number of hospital stays associated with these pathologies is increasing. The geographical variation in prevalence, and the presented trends of incidence require reorganization in the health care system to afford optimal medical care in the upcoming years.

## Figures and Tables

**Table 1:** Analyzed codes of salivary gland diagnosis according to the ICD-10 classification, with descriptions and division into three types of pathologies: malignant, benign, and non-neoplastic.

ICD-10 Code	Description	Group of Pathologies
C07	Malignant neoplasm of parotid gland	Malignant neoplasms
C08	Malignant neoplasm of other and unspecified major salivary glands	
C08.0	Submandibular gland	
C08.1	Sublingual gland	
C08.8	Overlapping lesion of major salivary glands	
C08.9	Major salivary gland, unspecified	
D11	Benign neoplasm of major salivary glands	Benign neoplasms
D11.0	Parotid gland	
D11.7	Other major salivary glands	
D11.9	Major salivary gland, unspecified	
K11	Diseases of salivary glands	Non-neoplastic
K11.0	Atrophy of salivary gland	
K11.1	Hypertrophy of salivary gland	
K11.2	Sialadenitis	
K11.3	Abscess of salivary gland	
K11.4	Fistula of salivary gland	
K11.5	Sialolithiasis	
K11.6	Mucocele of salivary gland	
K11.7	Disturbances of salivary secretion	
K11.8	Other diseases of the salivary glands	
K11.9	Disease of salivary gland, unspecified	

**Table 2:** Structure of new cases of salivary gland pathologies between 2010 and 2019 in Poland.

Salivary Gland Pathologies		2010	2011	2012	2013	2014	2015	2016	2017	2018	2019	Total	Percentage of Incidence
Number of new cases according to type of pathology													
Non-neoplastic lesions		21,004	19,632	19,128	20,559	21,280	19,718	20,399	19,714	18,019	17,691	197,144	85.50%
Benign neoplasms		2080	2273	2244	2634	2812	3110	2579	2791	2955	3123	26,601	11.53%
Malignant neoplasms		847	807	812	778	732	737	557	533	485	556	6844	2.97%
Incidence per 100,000 adult inhabitants													
Non-neoplastic lesions		54.514	50.941	49.64	53.406	55.303	51.299	53.077	51.294	46.911	46.091	51.25	
Benign neoplasms		5.398	5.898	5.824	6.842	7.308	8.091	6.71	7.262	7.693	8.137	6.915	
Malignant neoplasms		2.198	2.094	2.107	2.021	1.902	1.917	1.449	1.387	1.263	1.449	1.779	
All pathologies of the salivary glands		62.11	58.933	57.571	62.269	64.514	61.308	61.236	59.942	55.867	55.676	59.944	
Age of patients according to the type of pathology (mean age ± SD)													
Non-neoplastic lesions		48.19 ± 20.48	49 ± 24.74	49.11 ± 20.54	49.89 ± 20.29	50.22 ± 20.47	50.21 ± 20.71	50.92 ± 20.67	51.19 ± 20.57	51.59 ± 20.98	51.93 ± 21.09	50.23 ± 21.05	
Benign neoplasms		52.77 ± 17.09	54.09 ± 17.42	54.1 ± 17.5	54.61 ± 17.19	54.81 ± 17.51	55.91 ± 17.01	56.32 ± 17.26	55.98 ± 16.85	57.25 ± 16.84	56.86 ± 16.81	55.27 ± 17.15	
Malignant neoplasms		61.04 ± 16.39	61.23 ± 15.92	60.42 ± 15.97	60.15 ± 16.95	63.38 ± 16.91	63.06 ± 15.77	63.07 ± 15.8	62.28 ± 16.96	64.5 ± 14.97	62.84 ± 16.97	62.2 ± 16.26	
Gender structure of patients according to type of pathology													
Non-neoplastic lesions	Men (%)	38.73%	38.98%	38.31%	38.80%	38.53%	38.50%	38.93%	39.53%	38.73%	38.92%	38.80%	
	Women (%)	61.27%	61.02%	61.69%	61.20%	61.47%	61.50%	61.07%	60.47%	61.27%	61.08%	61.20%	
Benign neoplasms	Men (%)	43.32%	44.21%	44.83%	45.52%	43.81%	45.98%	45.72%	45.90%	44.20%	46.30%	44.98%	
	Women (%)	56.68%	55.79%	55.17%	54.48%	56.19%	54.02%	54.28%	54.10%	55.80%	53.70%	55.02%	
Malignant neoplasms	Men (%)	58.44%	55.27%	54.93%	60.93%	53.69%	54.55%	55.30%	54.60%	59.38%	59.89%	56.70%	
	Women (%)	41.56%	44.73%	45.07%	39.07%	46.31%	45.45%	44.70%	45.40%	40.62%	40.11%	43.30%	

**Table 3:** Number of inpatient and outpatient services among patients with non-neoplastic, benign, and malignant salivary gland lesions in 2010–2019 in Poland (SD—standard deviation).

Salivary Gland Pathologies	2010	2011	2012	2013	2014	2015	2016	2017	2018	2019	Total	Average Number of Services per Patient (± SD)
Number of outpatient consultations												
Non-neoplastic lesions	39,765	37,808	38,217	42,616	44,267	42,634	43,050	42,366	39,023	39,249	408,995	2.07 ± 0.11
Benign neoplasms	6411	7506	7880	9445	10,361	11,426	11,951	13,237	13,435	14,803	106,455	4 ± 0.64
Malignant neoplasms	6684	6280	6666	7491	7599	7823	8271	8253	8212	8612	75,891	11.09 ± 3.58
Number of hospital stays												
Non-neoplastic lesions	5786	5781	6012	6390	7437	11,786	12,807	14,121	15,253	15,073	100,446	0.51 ± 0.24
Benign neoplasms	1800	1734	1808	1844	2180	2612	3104	3080	4153	5076	27,391	1.03 ± 0.31
Malignant neoplasms	1933	1836	1197	911	1233	1162	1589	1596	1721	2191	15,369	2.25 ± 0.93

## Data Availability

The National Health Fund Registry data are available at http://www.nfz.gov.pl (accessed on 30 June 2021).

## Supplementary Materials

The following are available online at https://www.mdpi.com/article/10.3390/ijerph19010179/s1, Table S1: The detailed number of newly diagnosed salivary glands pathologies with respect to all analyzed ICD-10 codes between 2010 and 2019 in Poland.
