# Supplementary material for: The Epidemiology of Salivary Glands Pathologies in Adult Population over 10 Years in Poland—Cohort Study
Source: Int J Environ Res Public Health. 2021 Dec 24;19(1):179. doi: 10.3390/ijerph19010179 (PMC8750634; doi:10.3390/ijerph19010179)
Supplement: Supplementary file 1 [file ijerph-19-00179-s001.zip › ijerph-1422323-supplementary.pdf]

Supplementary Materials

Table S1. The detailed number of newly diagnosed salivary glands pathologies with respect to all analyzed ICD 10 codes between 2010 and 2019 in Poland.

ICD-10	2010	2011	2012	2013	2014	2015	2016	2017	2018	2019	Total
Malignant neoplasms											
**C07**	535	554	555	534	485	474	341	326	299	366	4469
**C08**	215	158	135	129	138	119	106	74	82	92	1248
**C08.0**	54	45	62	62	60	80	54	61	49	51	578
**C08.1**	4	2	5	4	7	4	5	4	1	6	42
**C08.8**	8	16	24	23	17	26	16	27	27	18	202
**C08.9**	31	32	31	26	25	34	35	41	27	23	305
Benign neoplasms											
**D11**	1235	1271	1189	1275	1328	1362	957	1074	1001	1184	11876
**D11.0**	685	787	848	1147	1240	1487	1338	1445	1624	1658	12259
**D11.7**	77	105	118	139	128	159	177	172	213	149	1437
**D11.9**	83	110	89	73	116	102	107	100	117	132	1029
Noncancerous diseases											
**K11**	16920	14748	12697	13518	13810	12534	12118	11666	10535	10299	128845
**K11.0**	564	503	601	576	548	359	349	276	239	214	4229
**K11.1**	128	197	250	223	252	223	202	188	164	150	1977
**K11.2**	1391	1710	2304	2470	2626	2567	3007	2880	2745	2602	24302
**K11.3**	104	98	112	120	115	134	121	116	124	105	1149
**K11.4**	28	30	30	31	27	31	30	26	17	18	268
**K11.5**	737	959	1141	1300	1370	1313	1481	1387	1288	1289	12265
**K11.6**	269	270	308	292	366	426	383	375	354	373	3416
**K11.7**	44	56	75	121	153	133	125	137	153	239	1236
**K11.8**	463	613	954	1139	1190	1234	1533	1528	1346	1319	11319
**K11.9**	356	448	656	769	823	764	1050	1135	1054	1083	8138
**Total**	25941	24723	24196	25984	26838	25580	25551	25055	23477	23389	230589
